# Recent Advances in Computational Protocols Addressing Intrinsically Disordered Proteins

**DOI:** 10.3390/biom9040146

**Published:** 2019-04-11

**Authors:** Supriyo Bhattacharya, Xingcheng Lin

**Affiliations:** 1 Division of Research Informatics, Beckman Research Institute at City of Hope National Medical Center, Duarte, CA 91010, USA; 2 Center for Theoretical Biological Physics, Rice University, Houston, TX 77030, USA; xclin@mit.edu; 3 Department of Chemistry, Massachusetts Institute of Technology, Cambridge, MA 02139, USA

**Keywords:** intrinsically disordered protein, conformational ensemble, nuclear magnetic resonance, replica exchange molecular dynamics, drug design

## Abstract

Intrinsically disordered proteins (IDP) are abundant in the human genome and have recently emerged as major therapeutic targets for various diseases. Unlike traditional proteins that adopt a definitive structure, IDPs in free solution are disordered and exist as an ensemble of conformations. This enables the IDPs to signal through multiple signaling pathways and serve as scaffolds for multi-protein complexes. The challenge in studying IDPs experimentally stems from their disordered nature. Nuclear magnetic resonance (NMR), circular dichroism, small angle X-ray scattering, and single molecule Förster resonance energy transfer (FRET) can give the local structural information and overall dimension of IDPs, but seldom provide a unified picture of the whole protein. To understand the conformational dynamics of IDPs and how their structural ensembles recognize multiple binding partners and small molecule inhibitors, knowledge-based and physics-based sampling techniques are utilized in-silico, guided by experimental structural data. However, efficient sampling of the IDP conformational ensemble requires traversing the numerous degrees of freedom in the IDP energy landscape, as well as force-fields that accurately model the protein and solvent interactions. In this review, we have provided an overview of the current state of computational methods for studying IDP structure and dynamics and discussed the major challenges faced in this field.

## 1. Introduction

Intrinsically disordered proteins (IDPs) have emerged as an important class of biomolecules that are involved in a variety of cellular functions, ranging from signaling to gene expression, chaperoning, and cellular transport [1]. These proteins lack a definite folded structure and adopt an ensemble of conformations in their physiological environment. Their disordered nature is essential for their biological function, as has been discussed in many recent reviews [1,2,3,4]. The IDP function is regulated through a fine balance of alternative splicing, post-translational modifications, expression level, and duration of presence in the cell [2]. Due to their dynamic nature, many IDPs can interact with multiple proteins with low affinity but high specificity, enabling them to act as hubs in signaling networks, or adapters for multi-protein scaffolds [1]. The low affinity of certain IDPs facilitates fast disengagement from signaling partners, which could be advantageous for rapid shutdown and switching of signaling, allowing better control over the cellular machinery [5]. Conversely, IDPs have also been found to interact with their partner proteins with ultra-high affinity, while maintaining structural disorder and flexibility in the bound state. [6] Dysregulation of IDP function has been linked to multiple cancers, as well as diabetes, cardiovascular, and neurodegenerative diseases [4]. IDP conformational dynamics has been proposed to facilitate phenotypic switching in stem cells and malignancy, without involving mutagenesis [7]. Therefore, IDPs form an important class of therapeutic targets, whose novel mechanisms and dynamic properties open new ways of regulating cellular function through clinical intervention [8].

In past decades, the emergence of IDPs has changed the traditional structure-function paradigm of folded proteins, which posits that sequence dictates the native state (an ensemble of closely related structures), which in turn dictates function. In contrast, the sequence of an IDP encodes an ensemble of structurally diverse conformations, which exchange in the picosecond to millisecond timescale under physiological conditions [9]. The biological function of the IDP is governed by the nature of this ensemble as well as the associated dynamics of conformational exchange. Experimentally, the IDPs are studied using a combination of techniques, including nuclear magnetic resonance (NMR), small angle X-ray scattering (SAXS), circular dichroism (CD), and single molecule spectroscopy [3]. These methods provide information about local residue contacts and side chain orientations, secondary structure content, as well as the dynamics and lifetime of such contacts. Such information, however, comes as statistical averages of entire ensembles, and do not give information about individual conformations in the ensemble. The structural heterogeneities of IDPs require further development of analytical/computational tools for an accurate description of their statistical properties. Previous major efforts include using modified polymer models that are more relevant for the dynamics of IDPs [10,11,12], testing and improving on the solvent model [13,14], and the development of new tools for analyses of experimental data [15,16]. An alternative approach would be to combine experimental data with statistical structural models and physics-based simulations of protein dynamics to generate the molecular models that account for the heterogeneous IDP ensemble [17]. Besides this, coarse grain and enhanced sampling simulations can give valuable insights into the oligomerization and interaction of IDPs with partner proteins. However, the sheer time and length scales associated with IDP dynamics pose serious challenges to the application of computational methods, as is evident from the recent literature [18,19]. Needless to say, there is an acute demand in the scientific community for precise atomistic models of IDPs for gaining functional insights as well as designing therapeutic agents inhibiting their functions. In this review, we discuss the various computational protocols that are relevant to IDP research. Such protocols range from structure prediction of IDPs, studying their dynamic behavior and mechanisms of partner protein interaction, to small molecule inhibitor design. In the end, we summarize the challenges involved in applying these methods and possible future directions in this field.

## 2. Energy Landscape of Intrinsically Disordered Proteins

**Figure 1:**
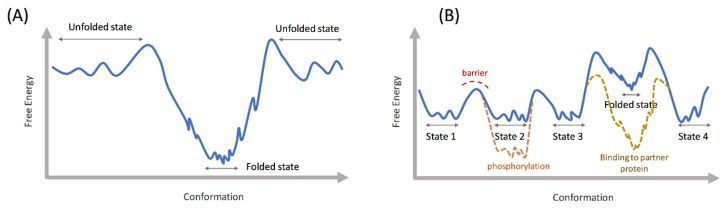
(**A**) Schematic free energy landscape of a folded protein; the folded state is comprised of an ensemble of structurally related states; (**B**) hypothetical intrinsically disordered protein (IDP) landscape showing four disordered (unfolded) states populated in free solution, separated by shallow energy barriers for rapid exchange; the multiple shallow minima in the disordered state free energy landscape indicate high structural flexibility; post translational modifications such as phosphorylation can stabilize one of the states over the others; a folded state is also present in the energy landscape, but only stabilized upon binding to a partner protein.

All proteins exhibit complex dynamic behavior, ranging from high frequency vibrations to slower local perturbations (picoseconds to microseconds), to even slower domain motions (microseconds to seconds). In a landmark paper, Wolynes and coworkers connected the dynamics, conformational sampling, and folding of proteins to the concept of free energy landscape [20]. Well folded proteins that adopt a definite three-dimensional (3D) structure show a funnel like landscape, where the initially formed secondary structure elements cooperatively fold into a tertiary structure stabilized by native contacts. The sequence of such a protein contains strategically placed hydrophobic residues, which in the folded state come together and form a hydrophobic core by efficient expulsion of water. An energy landscape describing such a process is relatively smooth with less frustration (as shown in the schematic in Figure 1A), although misfolded states and intermediate stable states could be populated in the landscape. In protein folding, frustration refers to the hindrance in progressing down the folding funnel, due to a lack of cooperativity in forming increasingly stable contacts. In contrast with a folded protein, the energy landscape of an IDP is proposed to be rugged (Figure 1B), where multiple states are separated by shallow energy barriers to facilitate exchange between the states [21,22,23]. Secondary structure elements may be present in free solution, but they do not cooperatively contact each other to fold into a stable structure as in the case of folded proteins. A reasonable explanation for the IDPs’ resistance to folding is the relative absence of hydrophobic amino acids in their sequences compared to folded proteins [3]. These amino acids are critical for forming the protein core and their absence stabilizes the unfolded states. In some IDPs, the abundance of disorder promoting residues, such as glycine and proline, inhibit the formation of stable helices and prevent folding tertiary structures. IDPs thus exist as an ensemble of extended or partially folded states in free solution with frequent exchange between these states. This rapid exchange prevents the detection of any single state in NMR or circular dichroism experiments [21]. Each of the IDP conformations could be functionally relevant in recognizing specific partner proteins for signaling or arranging a scaffold. In this regard, IDPs are distinct from random polypeptide sequences [21] because, although disordered, the IDP conformations could strategically orient specific recognition motifs such as phosphorylation sites to facilitate interaction with partner proteins. Post-translational modifications such as phosphorylation play a critical role in reshaping the conformational landscape of IDPs, preparing them for specific binding and signaling events [24,25]. Although IDPs mostly remain disordered in free solution, one or multiple intermediate states with ordered motifs may still be present in the energy landscape [26,27], albeit at a higher (worse) free energy than the disordered conformations [28]. In certain IDPs, such as the Aβ40 peptide, these partially ordered states become more predominant at a higher temperature, indicating their presence in the free energy landscape at a higher free energy than the unfolded states [28]. The presence of such partially ordered states in the IDP energy landscapes has been shown using NMR and circular dichroism, as well as enhanced sampling molecular dynamics (MD) methods [28,29,30]. The partially ordered states may be lowered in energy and stabilized upon binding to partner proteins or aggregation (e.g., amyloid fibrils), as shown in Figure 1B [28,31]. Also, the presence of diverse partially ordered states in the free energy landscape may facilitate interaction with multiple partner proteins, which is a hallmark of IDPs.

## 3. Structure Prediction and Conformational Dynamics

### 3.1. Conformational Selection Based Methods

**Figure 2:**
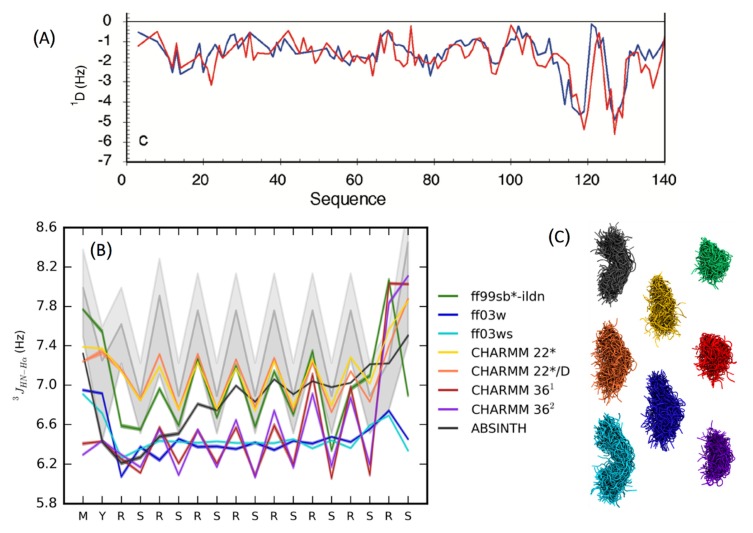
(**A**) Comparison between experimental (blue) and simulated ^1^D_NH_ residual dipolar coupling (RDCs, red) from ensemble of α-synuclein generated by flexible-meccano and ASTEROIDS (a selection tool for ensemble representations of intrinsically disordered states); reproduced with permission from [95]; (**B**) comparison of experimental J coupling data from a disordered peptide with those calculated from ensembles obtained from replica exchange molecular dynamics (REMD) simulations with various force fields; The experimental ^3^J_HN-Hα_ couplings are shown in gray shading (**C**) ensembles obtained from various force fields in cartoon representation; reproduced with permission from [69].

Computational structure prediction for folded proteins suffers from the challenges of conformational sampling, due to the numerous degrees of freedom of the polypeptide chain, and the difficulty for a protein to run across thermodynamic barriers among a set of structurally close decoys within limited simulation time to identify the correct native ensemble. In contrast, IDPs have a larger set of “correct answers” with regard to structure—an ensemble of highly diverse conformations ranging from random coil to partially folded structures [32]. The challenge is in exhaustively sampling the set of thermodynamically relevant conformations under physiological conditions, that explain the available experimental structural data (e.g., NMR, SAXS). So far, several computational methods have been proposed that utilize experimental structural information to derive conformational ensembles of IDPs. These methods can be categorized into two groups: a) one that uses NMR, SAXS, or Förster resonance energy transfer (FRET) data to select the relevant conformations from a pool of previously generated structural ensemble, and b) one that uses structural restraints derived from experimental data to drive MD or Monte Carlo (MC) based sampling methods to generate the conformational ensembles [19,22]. The former category includes methods such as TraDES [33,34], flexible-meccano [35], ASTEROIDS [36], and ENSEMBLE [37]. Both TraDES and flexible-meccano use probability distributions of amino acid orientations from crystal structures to sample the IDP conformations. While flexible-meccano generates chain conformations by randomly selecting the Φ/ψ dihedral angles of the residues from the distributions derived from the crystal structure database, TraDES grows the chain one residue at a time, using a Markov process. Both methods use specialized algorithms to accelerate the conformational sampling, by using fast detection of steric clashes between atoms and avoiding slow force field-based energy calculations. The conformational sampling can also be further guided by utilizing secondary structure prediction or other user-defined criteria. Such a conformational search is by no means exhaustive and becomes more challenging for larger proteins. Moreover, electrostatics and solvent-protein interactions are ignored, which may lead to unrealistic protein conformations in the ensemble, for example with water exposed hydrophobic segments. Conformation generation programs like TraDES and flexible-meccano are often used in conjunction with conformation selection programs such as ASTEROIDS and ENSEMBLE to generate a subset of conformers that fit available experimental data, such as chemical shifts, NOE (nuclear overhauser effect), RDC (residual dipolar coupling), PRE (paramagnetic relaxation enhancement), SAXS, and hydrodynamic radius (Figure 2A). Starting from an initial pool of over 100,000 chain conformations, ENSEMBLE selects a random subset of 5000 or less conformers and calculates the experimental properties, such as RDC, by averaging over the ensemble [37]. It then tests the agreement of the calculated properties to the observed experimental data. The program then adds more conformations to the set and eliminates existing conformations using a Monte Carlo scheme, coupled with simulated annealing. The process converges when further iterations do not improve the agreement with the observed data. ASTEROIDS uses a genetic algorithm to evolve an initial pool of conformations to minimize a fitness function based on the difference between calculated and observed experimental parameters. Such an ensemble filtering approach, when combined with maximum likelihood algorithms, can mitigate the problem of overfitting [38,39].

### 3.2. Molecular Dynamics Based Methods

In contrast to the above methods that attempt to reproduce the experimentally observed IDP ensemble by random sampling of polypeptide conformations, MD based methods perform the conformational sampling using molecular dynamics algorithms, which simulate the dynamic behavior of IDPs under physiological conditions in a solvent environment. Due to the computationally intensive energy functions and the difficulty to cross over energy barriers, most MD based methods are orders of magnitude slower than random sampling based algorithms. However, the benefit of MD based methods is in the insights gained into the protein dynamics, such as exchange rates between different conformations, persistence of specific inter-residue contacts, or the interactions of solvent and ions with the protein. To facilitate sampling of the rugged conformational landscapes of IDPs, MD algorithms are usually coupled with enhanced sampling methods (also known as generalized-ensemble algorithms [29,40]), such as replica exchange (REMD) [41], bias exchange metadynamics (BEMD) [42], and temperature cool walking (TCW) [29,43]. In REMD, multiple MD simulations (replicas) of the same system are performed in parallel at different temperatures, with frequent exchange of operating temperatures between the replicas [41]. In TCW, the simulations are performed at two different temperatures, the target temperature and a higher temperature with transitions between the two temperatures facilitated by simulated annealing. In both methods, by temporarily raising the temperature of a replica, the system is able to overcome energy barriers that would have restricted sampling, had the MD been performed at a single temperature. The exchange probabilities are selected such that a thermodynamically consistent structural ensemble is obtained at the target temperature. The advantage of TWC over REMD is the lower computational resource requirement. While in REMD, 30 or more separate simulations are often required to cover the desired temperature range, but only two simulations are necessary in TWC, although the transition between the two temperatures is computationally demanding due to the use of simulated annealing (for a comparison between the two methods, please see [29]). In BEMD [42], multiple simulations (replicas) are performed at the same temperature, but with time-dependent biasing potentials along different sets of collective variables for each replica, with frequent exchanges of the biasing potentials between the replicas. Bias exchange metadynamics can be computationally efficient, since fewer replicas are required as compared to REMD. However, as with standard metadynamics simulations, the choice of the collective variables is crucial for proper sampling of the conformational space. Using REMD, Patel et al. compared the disordered conformational ensembles of two heat shock proteins, explaining their difference in efficiency for binding to unfolding proteins at higher temperatures [44]. The BEMD has been used to map the conformational landscapes of several IDPs, such as the Aβ40 peptide involved in Alzeimer’s disease and the islet amyloid polypeptide involved in type 2 diabetes [28,45]. Temperature cool walking was used to study the effect of paramagnetic spin labels on the conformational ensembles of IDPs [46]. In relation to the IDP ensemble, the structures themselves and the rates of transition between these structures, which govern the kinetics, are of equal importance. A change in environment, such as pH and temperature and post translation modifications, can affect these rates and hence the biological function of the IDPs. With the advent of fast GPU (graphical processing unit) powered computing networks, it is now possible to map transition rates among various equilibrium states in the protein landscape, using methods such as Markov state model (MSM) analysis [47]. The MSM uses numerous independent MD trajectories to calculate the equilibrium states and the rates of transition between these states. However, due to the numerous degrees of freedom in the IDP landscape, it is still challenging to apply MSM to calculate transition rates in IDPs. Using the distributed computing network GPUGRID.net [48], Stanley et al. developed a MSM for the kinase-inducible domain (KID) of the transcription factor CREB (cAMP response element-binding protein), and showed that phosphorylation slows down the conformational kinetics and facilitates binding with partner proteins [49]. Markov state model was also used to map the conformational landscape of the hIAPP peptide involved in fibril formation in type II diabetes [30]. Alternatively, exhaustive simulations of protein dynamics using ASIC hardwares such as Anton [50,51] have proved successful in folding proteins and studying protein dynamics over recent years [52]. The applications of Anton in studying IDPs provide another avenue for generating and understanding their heterogeneous structural ensembles [32]. 

One method which is promising for simulating IDP dynamics is generalized Newton–Euler inverse mass operator (GNEIMO) torsional dynamics [53,54,55]. By performing protein dynamics in the low frequency torsional degrees of freedom (DOF) while freezing the high frequency bond and angle vibrations via constraints, GNEIMO is able to simulate long timescale transitions that are difficult to achieve in all-atom Cartesian MD [56]. Besides the bonds and angles, selective torsional DOFs can be constrained as well, depending on the system of interest. Thus, given a protein with both disordered and folded regions, for instance, GNEIMO can simulate the conformational dynamics of the disordered domain in the environment of the relatively static folded domain, while keeping the backbone (and core sidechains) of the folded domain frozen. Constraining the uninteresting DOFs will lead to accelerated sampling and a higher chance of simulating long timescale transitions in the disordered region [55,56]. The GNEIMO employs specialized algorithms that are designed to preserve accurate dynamics with constrained DOFs, without introducing artifacts [55,57]. The GNEIMO, combined with temperature replica exchange, was used to simulate the dynamics of the flexible regions of fasciculin and calmodulin [56]. During dynamics, two of the experimentally verified conformational states of fasciculin were sampled by GNEIMO. For calmodulin, GNEIMO achieved the transition between the calcium bound and unbound conformations. Such conformational transitions were achieved in all-atom Cartesian MD by using biasing potentials [58], or using an united atom Gõ model [59].

The above examples demonstrate that the MD based methods, including both the enhanced sampling and brute force techniques, can give valuable insight into the conformational equilibria and the associated kinetics of IDPs. Intrinsically disordered proteins typically show a wide range of motion, from pico- to microseconds and longer. The rates/correlation times in these different temporal regimes, along with the flexibility of the protein domains (represented by the order parameters), can be obtained by deconvoluting the experimental data from NMR relaxation and fluorescence correlation spectroscopy (FRET-FCS) [60]. The question remains whether MD simulations can accurately reproduce the dynamic behavior of IDPs as reported by these experimental methods. In recent years, several works have compared the quantitative dynamics information of IDPs obtained from the experiments to those from the simulations. Tryptophan fluorescence quenching rates obtained with several model (disordered) peptides were found to be in agreement with microsecond scale MD simulations [61]. This work also addressed the effect of force field and solvent viscosity on the rate of inter-residue contact formation and diffusivity, which govern dynamic behavior. Using FRET-FCS, Soranno et al. [62] measured the relaxation time for inter-termini distance in the unfolded protein L, which agreed with the quantity calculated from microsecond long MD simulations on the ANTON supercomputer by Piana et al. [14]. The MD simulations were performed using an improved water model (TIP4P-D) to better reproduce the extended ensembles of IDPs. Using three different techniques (high-field ^15^N spin relaxation, low-field ^1^H relaxometry, nanosecond fluorescence correlation spectroscopy), Rezaei-Ghaleh et al. monitored the conformational dynamics of α-synuclein at different timescales (picoseconds to nanoseconds to 10s of nanoseconds) [63]. These timescales were compared to the equivalent parameters calculated from a 16 μs MD trajectory of α-synuclein in explicit water [14]. While the relaxation rates obtained from the low field ^1^H relaxometry and nanosecond fluorescence correlation spectroscopy were in agreement with the values obtained from MD, the relaxation times obtained from ^15^N spin relaxation were over-predicted. Also, compared to the order parameters from NMR, MD showed relatively restrictive backbone motion. To match the order parameter values from MD to those from NMR, the authors had to apply a scaling factor to the MD time axis. The results from these studies indicate that the MD simulations, with the appropriate force field and simulation length, can provide quantitative insights into IDP dynamics. Nevertheless, the discrepancies with experiments depending on the timescale of motion highlights the complex scenario with applying MD methods in studying IDP dynamics. This also impresses upon the need for developing accurate protein and solvent force fields that capture realistic dynamics as well as ensemble properties.

### 3.3. Force-Field Development for Intrisically Disordered Proteins

For MD based methods, accuracy of the underlying force field is a key determinant of the reliability of the obtained results. From the time when protein crystallography was first introduced in the 1950s, the general paradigm of protein function has rested with its folded structure, and most efforts were originally spent in understanding and predicting the native state of globular proteins. Early force field development focused on stabilizing the protein crystal structures, with less attention being payed to the unfolded state. As a result, these force fields generated conformations with overpopulated secondary structure elements (i.e., alpha helices and beta sheets) [18]. However, such inaccuracies have been largely corrected in the recent versions of these force fields by modifying the protein backbone dihedral parameters, and validating against experimental NMR and SAXS data [64,65,66,67]. The other issue with many general-purpose force fields is the over-stabilization of collapsed, molten globule like states compared to extended states. Both these issues are especially relevant while studying IDP structural ensembles, which lack extensive secondary structures and are less compact than folded proteins. The accuracy of various force fields has been widely discussed in the literature [18,68]. Rauscher et al. did a detailed comparison of CHARMM, AMBER, and OPLS force fields in conjunction with several water models (TIP3P, TIP4P, and implicit solvent) [69] (Figure 2B,C). This study highlighted the inherent bias of all-atom force fields towards certain secondary structures, for example, the propensity of CHARMM36 to overpopulate left-handed α-helices in disordered peptides. Also evident was the stabilization of over-compact structures by CHARMM36 and FF99SB*-ILDN (Figure 2C). Corrections to such biases or deficiencies have been partially addressed in revisions to the existing force fields that are targeted specifically towards simulating IDPs. Examples include force fields from the AMBER family, such as FF03* and FF99SB* [65], and CHARMM family force fields CHARMM36m [67] and CHARMM22* [70]. Since solvent–protein interactions play an important role in the structural propensity of IDPs, it is desirable, alongside the protein backbone parameters, to optimize the protein–water interaction parameters as well. Such modifications produced the AMBER FF03ws [71] and, recently, the A99SB-disp [32] force fields. The latter was developed through a combined optimization of protein dihedral parameters, hydrogen-bonding potentials and partial charges alongside water van der Waals parameters to reproduce the structural properties of both folded and disordered proteins. Other efforts in the development of force fields for IDPs include RSFF [72,73], KBFF [74], CUFIX [75], and ff14idp [76].

### 3.4. Coarse Grain and Multiscale Methods

Due to the computational expense of all-atom MD simulations in explicit water, coarse-grain protein and solvent models are viable alternatives to study the conformational ensemble of IDPs. In the coarse grain description of the polypeptide chain, several atoms are combined into one moiety, with a similar treatment applied to the solvent atoms. A special force field function is developed to model the interaction between the different coarse grain moieties in a way that reproduces protein-specific behavior. An alternative approach to coarse-graining is the implicit solvation model, where the explicit solvent molecules are replaced by a mean field approximation, while the protein is kept fully atomistic. Implicit solvation models can reproduce aspects of the protein–solvent interaction, such as cavity penalty, electrostatic screening, and nonpolar burial. However, the detailed behavior of individual solvent molecules at the protein/bulk water interface, including water mediated hydrogen bonds, are not retrieved in such models. The use of coarse grain models reduces the number of particles in the simulation box, leading to a significant gain in computational performance and enhanced sampling of the conformational space. These models can be potentially used to simulate long timescale processes, such as crowding and self-assembly of IDPs, which are normally beyond the reach of all-atom MD based methods. Implicit solvation methods such as Generalized Born [77] have been widely used to predict structures of folded proteins [78,79]. However, their application to IDPs [23,80] is limited by 1) the computational cost involved in frequently calculating the effective Born radii in a rapidly changing protein environment (more so than in folded proteins) [81], and 2) over-stabilization of secondary structural elements and compact ensembles [31,82]. The latter problem was addressed by Lee et al. by optimizing the GBMV2 (Generalized Born using molecular volume) implicit solvation model [83], in conjunction with the CHARMM36 protein force field. The improved GBMV2 model predicted fewer compact ensembles, in agreement with the experimental structural properties of several IDPs [82]. One of the implicit solvation models that has shown success in the IDP field is the ABSINTH model (self-assembly of biomolecules studied by an implicit, novel, and tunable Hamiltonian) by Vitalis and Pappu [81,84]. The ABSINTH builds and improves upon the EEF1 (effective energy function) solvent model [85] that calculates the solvation free energy of the protein as a sum of individual backbone and sidechain components. The solvation energies of the individual components are obtained from experimental solvation free energies of small molecules that resemble the protein components and are weighted by their solvent exposure during simulation. The ABSINTH has been used to explore the effect of temperature and charged amino acid distribution in the protein sequence on the ensemble properties of IDPs [86,87].

Alongside the implicit solvation models that approximate the electrostatic contribution of the solvent environment towards fully atomistic protein structures, advancements have been made towards modeling the polypeptide chain in a coarse grain form. The associative-memory, water-mediated, structure, and energy model (AWSEM) [88,89], which was originally developed to study globular protein folding, has been successfully modified for application to IDPs [90]. The AWSEM incorporates a coarse grain potential function comprising of terms that model hydrophobic, hydrogen bond, and water mediated interactions, as well as terms to control secondary structure formation and local tertiary interactions, both of which can be obtained from database or experimental/computational studies. A recent computational study applied AWSEM to study a cancer-related IDP, prostate-associated gene 4 (PAGE4), and successfully reproduced the structural switch of this molecule induced by different levels of phosphorylation [91]. Using the modified coarse grain protein model PLUM [92], Rutter et al. reproduced the secondary structures and disorder propensities of amino acids in the N terminus of the n16 peptide involved in biomineralization [93]. The predicted conformations showed agreement with all-atom MD simulations. These coarse grain simulations also successfully distinguished the dimerization propensity of the n16 peptide with its mutant which fails to dimerize, and in a later paper, the aggregation behavior of multiple peptides [94]. Such developments give hope for increasing the scope of MD simulations beyond the conformational sampling of single protein chains and enable simulation of complex processes such as aggregation, self-assembly, and vesicle formation involving IDPs.

## 4. Protein–Protein Interaction Involving Intrinsically Disordered Proteins

Due to their conformational diversity, IDPs interact with multiple partner proteins, DNA, and RNA, leading to signaling, transport, endocytosis, gene expression, and fibril formation. The association of IDPs ranges from homo and hetero-dimerization to aggregation involving hundreds of monomers. Many IDPs adopt folded structures upon binding to their partners and the same IDP can adopt multiple folded structures depending on its binding partner [5,96]. Alternatively, disorder can be retained upon binding, in the so called fuzzy complexes [97,98]. Besides proteins, IDPs are also involved in interactions with ligands, nucleic acids and membranes, details of which can be found in several reviews [8,99,100]. 

### 4.1. Disorder Retained upon Binding: Fuzzy Complexes

**Figure 3:**
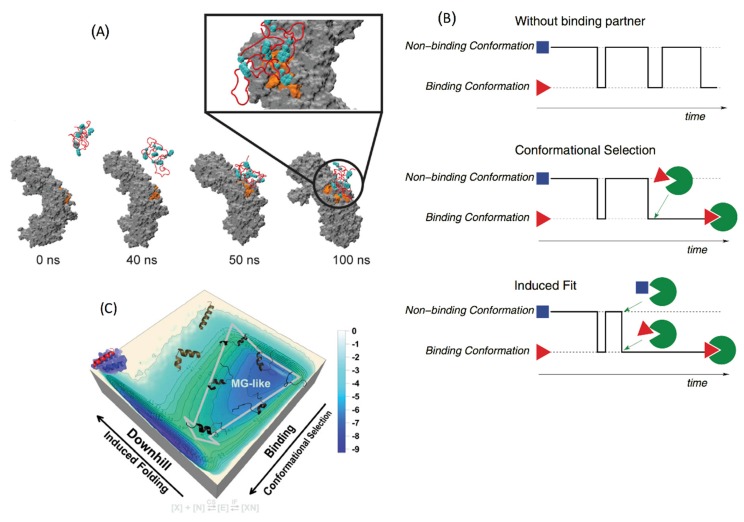
(**A**) Formation of a fast binding fuzzy complex between nucleoporin (red cartoon) and importinβ (grey surface), simulated using an all-atom molecular dynamics (MD) simulation; the binding sites on importin β and nucleoporin are colored in orange and cyan respectively; reproduced with permission from [101] (https://doi.org/10.1016/j.cell.2015.09.047) under the terms of the Creative Commons Attribution License (CC BY); http://creativecommons.org/licenses/by/4.0/ (**B**) schematic demonstration of conformational selection and induced fit binding mechanisms; in the absence of binding partner, the IDP switches between the non-binding (blue) and binding (red) conformations; in conformational selection, the IDP binds to the partner protein in the binding conformation without any structural rearrangement; for induced fit binding, the IDP initially encounters the partner using the non-binding conformation, then adopts the binding conformation in presence of the partner; reproduced with permission from ref. [108] (**C**) coarse-grain free energy landscape showing a combined conformational selection and induced fit binding of a disordered C terminal segment of the measles virus nucleoprotein to the X domain of the measles virus phosphoprotein; reproduced with permission from ref. [103]; Copyright 2013 national Academy of Sciences.

Many IDPs are reported to form fuzzy complexes with proteins and nucleic acid polymers, whose importance in the biological processes has become increasingly clear in recent years [97,98]. The IDP involved in a fuzzy complex maintains its structural disorder upon binding to the partner protein. The binding is driven by increase or no loss in entropy and lowering of enthalpy via multiple weak protein–protein interactions [98]. In some cases, both the binding partners remain disordered upon binding, as in the case of histone H1 and prothymosin-α. Here, the binding is driven by long range electrostatic attraction, as opposed to specific inter-residue interactions [6]. As part of a combined theoretical and experimental work, Milles et al. studied the association between nucleoporins and the nuclear transporter importin β using all-atom MD simulations [101]. Out of ten independent simulations, four binding events were observed within 100 ns (Figure 3A), and the predicted binding site interactions agreed with the observed NMR ^1^H-^15^N HSQC (heteronuclear single quantum coherence) spectrum. The multiple binding events within a short simulation time is in line with the ultra-fast association rates of fuzzy complexes, which do not have the rate limiting step of structural rearrangement or folding upon binding. Also, fuzzy complex IDPs can bind to their partner proteins through multiple pathways, leading to fast binding. 

### 4.2. Conformational Selection versus Induced Fit

For IDPs that adopt folded structures upon binding to their partner proteins, two types of mechanisms have been proposed: (a) conformational selection and (b) induced fit [1,102]. In conformational selection, one of the preexisting folded monomer conformations binds to the partner protein, whereas in induced fit, the folding happens subsequent to binding (Figure 3B). Most IDPs are thought to follow a combination of the two mechanisms, where one of the partially folded metastable states binds to the partner protein (selection), followed by the rest of the folding (induced fit), which is facilitated by the environment of the other protein [103]. Such complex binding mechanisms often incorporate multiple steps with associated energy barriers and are typically beyond the scope of all-atom unbiased MD simulations in an explicit solvent environment. As a result, coarse grain simulations or all-atom biased simulations are employed where the interaction energy between different parts of the protein are modeled based on experimental structural data and physics-based potentials. Coarse grain models such as the modified Gõ-like models have been extensively used in studying the binding of IDPs [104,105,106,107,108]. The coarse-grained Gõ model was originally developed to study the folding of globular proteins [109]. It is based on the minimally frustrated energy landscape theory [110,111], which posits that foldable protein sequences have evolved to create a funnel shaped energy landscape, where only the native contacts are energetically favored and non-native contacts are not. Thus, the force field interactions in the Gõ model are chosen to stabilize the native state over non-native conformations, in order to approximate the protein free energy landscape. To adapt the Gõ model to study IDPs which sample multiple conformations instead of one native state, multiple topology based energy terms (each specific towards one conformation) can be combined together using exponential mixing, as described in [108]. The resulting potential function can describe an energy landscape with multiple local minima, separated by free energy barriers controlled by a mixing temperature. The parameters for modeling the energy function can be derived from the crystal and NMR structures of IDP complexes. Such a model, though simplistic compared to all-atom physics-based models, can be a powerful tool to study kinetics and free energy landscapes of IDP binding. Using a multistate Gõ model, Knott et al. studied the binding of the disordered nuclear coactivating binding domain (NCBD) of the CREB binding protein to the ACTR domain of p160 and the interferon regulatory factor IRF-3 (NCBD adopts different folded conformations upon binding to ACTR and IRF-3). The energy parameters of the Gõ model were derived from the crystal structures of the two bound complexes and the respective binding affinities. The authors showed that the binding of NCBD to both ACTR and IRF-3 follow an induced fit mechanism. In a separate work, Ganguly and coworkers showed that non-specific electrostatic interactions play a key role in orienting NCBD and ACTR towards the native interaction surface to facilitate fast binding [107]. Wang et al. developed a hybrid potential based on both physics based and native topology-based terms, which they used to study the mechanism of binding between the disordered measles virus nucleoprotein (MeV) and the phosphoprotein X domain (XD) [103]. The resulting binding free energy landscape showed that the initial binding of MeV proceeds via conformational selection of an α-helical monomer conformation, followed by induced fit that results in complete folding of MeV around XD (Figure 3C).

### 4.3. Liquid–Liquid Phase Separation and Aggregation

Due to their disordered and flexible nature, IDPs can exhibit a variety of complex thermodynamic states, involving multiple proteins, RNA (or DNA), small molecules, and ions [8]. One prominent feature of IDPs is their involvement in the formation of proteinaceous membrane-less organelles (PMLOs) [112]. These organelles, that are abundant in both eukaryotic and plant cells [113], are an essential part of the biological repertoire, being involved in sequestering enzymatic reactions and small molecules or RNA/DNA from the bulk cytoplasm [114]. Such organelles may also be involved in protein aggregation related neurodegenerative diseases [115]. The PMLOs are formed by the localized separation of a protein rich liquid phase (droplet) in equilibrium with a surrounding dilute phase, a phenomenon commonly termed as liquid–liquid phase separation (LLPS). In LLPS, the protein-rich phase shows liquid like properties such as low shear and surface wetting, and is distinct from protein aggregate rich organelles such as inclusion bodies [113]. The flexibility of the IDPs and the abundance of charged amino acids in their sequence are often cited as reasons for facilitating LLPS [112]. The IDP domains that participate in phase separation often consist of charged amino acid repeats (low complexity regions) that create multiple sites of interaction with neighboring proteins, facilitating condensate formation [8,113,116]. However, there are currently unanswered questions regarding the detailed mechanisms of IDPs in undergoing LLPS, and the role of amino acid composition and placement, presence of folded domains, temperature, pH, post translational modification, and concentration of different precursors such as RNA [112,116]. The problem of IDP phase separation is best addressed by coarse grain MD methods [117] and analytical and semi-analytical polymer theory [118], since the timescale of the slow diffusion controlled rearrangement of multiple IDP chains involved in phase separation is beyond the reach of all-atom MD simulations. See [119] for a complete review on coarse grain and multiscale simulations of the phase behavior of IDPs. 

Coarse grain models, such as lattice models, are simplistic but highly powerful tools to explore the phase behavior of polymers and surfactants [120,121]. In a lattice model, the simulation box is discretized into a grid, and the protein (and RNA) is modeled as a chain of beads occupying adjacent lattice sites. The beads of neighboring chains interact using a square well potential, where the interaction strength is determined by the knowledge of the individual components. The system is simulated by moving the beads to nearby lattice sites and accepting or rejecting the moves using Monte Carlo. Using both experiments and coarse-grain lattice Monte Carlo simulations, Boeynaems et al. provided insights into the formation of multi-layered PMLOs by IDPs, in the presence of RNA and polyanionic proteins [117]. The authors showed that RNA molecules lacking stable secondary structures facilitate the formation of LLPS droplets, whereas base pairing of RNA leads to (metastable) solid-like gels. In another work using continuum coarse grain models, Dignon et al. simulated the phase properties of two different IDPs (one of which is involved in fibril formation in amyotrophic lateral sclerosis (ALS)) [116]. Their coarse grain model included a single bead for every amino acid, interacting with each other through van der Waals and screened electrostatic interactions, whose parameters were optimized by comparing with experimental radii of gyration of the proteins of interest. The authors simulated the phase behavior of the proteins at different temperatures, thus constructing the complete phase diagram. They also discussed the effect of mutations and the presence of folded domains on the observed phase behavior. Lastly, analytical and semi-analytical polymer theories have been applied for modeling IDP phase behavior. Such theories come with different degrees of approximation and resolution, and they can capture salient features of IDP phase properties, such as the effects of amino acid pattern and chain length. For a recent work in this area, please see [122].

Several IDPs, for example the tau protein, amyloid β peptides, α-synuclein, and hIAPP, show a propensity to form high density, fibril like aggregates that are implicated in diabetes and neurodegenerative diseases like Alzheimer’s (AD) and Parkinson’s [123]. It has been proposed that transient secondary structure elements naturally present in these IDP ensembles can induce aggregation and fibril formation under overcrowded conditions [31]. Qiao et al. constructed the Markov State Model for the hIAPP peptide involved in amyloid fibril formation in type 2 diabetes through extensive MD simulations [30]. These simulations revealed metastable states in the energy landscape, which were shown to facilitate fibril formation. A well-studied IDP involved in fibril formation is the Aβ peptide. Using REMD simulations, Das et al. studied the conformational landscape of the wild type Aβ peptide and two of its mutants, the disease resistant A2T and A2V, which induces early onset Alzheimer’s [124]. While the A2T mutation inhibited the wild type population of β hairpin structures that promote aggregation, the A2V mutation preserved such conformations. Using the MD simulations, the authors were able to provide insights into the mechanisms behind such conformational shifts. In a separate study, REMD simulations were used to explain the binding mechanism of short peptides (based on the A2T and A2V mutants) to wild type Aβ peptide, that were designed to impede fibril formation [125]. The atomistic MD simulations provide insights into the aggregation process by analyzing the conformational propensity of isolated IDP monomers. Simulating the actual aggregation process, however, is beyond the scope of atomistic simulations, and would require coarse grain protein models. Examples of works in this area include [126,127]. 

## 5. Intrinsically Disordered Proteins as Therapeutic Targets

Due to the increasing evidence of IDPs being involved in various diseases and their abundance in the human genome [4,128], these proteins have emerged as promising drug targets. Many IDPs act as signaling hubs (central points in cellular signaling networks) and dysregulation of these hubs often has a significant effect on the cellular function. In diabetes and neurodegenerative diseases, aggregation of IDPs, such as hIAPP and tau-protein, lead to β cell death and amyloid fibril formation [123,129]. Thus, designing small molecules or peptides to bind to IDPs is a viable strategy to modulate signaling networks or prevent aggregation and reverse disease phenotypes. To date, the discovery of several drug candidates that bind to disordered regions of proteins reinforces this possibility [130,131,132,133,134,135]. However, some of the challenges involved in targeting IDPs for drug discovery are: 1) lack of stable binding pockets due to high inherent flexibility [135,136,137], 2) weak affinity of the binders due to transient interaction with the protein [128], and 3) potential lack of selectivity to the target IDP [128]. The suggested strategies for targeting IDPs with small molecules include stabilizing IDPs in their native disordered ensemble (to prevent aggregation), inhibiting interaction with specific binding partners, and allosteric modulation of protein conformation [136]. 

Experimental drug discovery relies on high throughput screening assays (HTS), which can be expensive and are limited by the number of compounds that can be screened (up to 100,000 compounds a day). Computational drug screening (high throughput virtual screening, HTVS) [138] can filter small molecule databases with more than a million compounds and significantly reduce the number of candidates to be tested in experimental assays, saving time and effort. However, most of the existing methods for computational drug discovery are designed to identify compounds that bind to deep, well-formed pockets in conventional folded proteins. The principles that help to distinguish binders from non-binders in these proteins are shape fitting, stable, energetically feasible protein–ligand interactions and effective shielding from the solvent upon binding. In contrast, IDPs adopt multiple conformations in free solution (instead of a single dominant one that can be targeted for designing a drug) and lack stable hydrophobic cores that would facilitate the formation of deep pockets. In fuzzy complexes, the protein–protein interface is comprised of many weak and dynamic inter-protein interactions, as opposed to a few stable hotspots. This makes it highly challenging to design small molecules using the established drug design principles to disrupt such interactions. However, IDPs can have relatively rigid hydrophobic pockets, while the rest of the structure remains flexible, as shown in the case of nuclear protein 1 (NUPR1). [132] Such pockets can be targeted for binding small molecules. Several small molecules have been successfully identified and confirmed to bind to disordered regions of proteins, for example the oncogenic transcription factor c-Myc, that disrupts its association with its partner protein Max [130,131]. The NMR nuclear Overhauser effect spectroscopy (NOESY) data showed that two separate binding sites exist in c-Myc, that bind two different small molecules. Multiple small molecule inhibitors have been identified for the IDP α-synuclein, which is a major target for Parkinson’s disease [133,134]. An analysis of 51 IDP structures from public databases showed that IDPs contain on average 50% more druggable pockets than folded proteins [139]. This evidence suggests that it is feasible to design small molecules to bind to IDPs and modulate their functions, potentially with high specificity [128]. However, new design principles are needed for identifying small molecule binders to IDPs, which have to be formulated based on mechanistic insights gained from studying existing examples. 

The binding sites and interactions between small molecules and their cognate IDP partners have been studied using NMR and molecular simulations. Using NMR guided docking and MD simulations, Neira et al. studied the binding of several small molecules to the IDP NUPR1 involved in pancreatic duct adenocarcinoma [132]. These compounds were found to bind to a hydrophobic region in the NUPR1 monomer that was relatively rigid compared to the rest of the structure. Thus, binding of the compounds didn’t significantly reduce the flexibility of the protein and minimized entropy loss. Additionally, they shielded hydrophobic residues such as Phe, Tyr, and Leu from water exposure, leading to favorable binding. A similar binding of small molecules to a hydrophobic/aromatic rich region in c-Myc was reported by Michel et al. [140]. Using BEMD simulations in explicit water, the authors compared the conformational ensemble of c-Myc in the presence and absence of the small molecule 10058-F4, followed by validation with NMR chemical shift data. 10058-F4 was shown to preferentially bind to several protein conformations that were less populated in the free solution. Upon the binding of 10058-F4, the equilibrium shifted towards the increased population of the ligand specific conformations. The preexistence of small molecule binding conformations in free monomer ensemble (although in fringe proportion) was shown in the flexible fusion peptide of HIV-1 using collective MD simulations [141]. These studies give some useful pointers towards targeting IDPs with small molecules: 1) the conformational ensemble of the ligand free IDP may already have conformations that are conducive to binding small molecules, 2) extensive conformational sampling using computational methods is capable of sampling these conformations, and 3) relatively rigid or hydrophobic domains on IDP surfaces could be potential hotspots for drug interaction, and this could serve as a signature for selecting the small molecule specific conformations from an ensemble. However, it may be advantageous to give priority to those conformations that are already highly populated in the ligand free ensemble. As mentioned in [141], targeting less populated IDP conformations for drug design could elicit a higher dose requirement of the resulting drug. 

**Figure 4:**
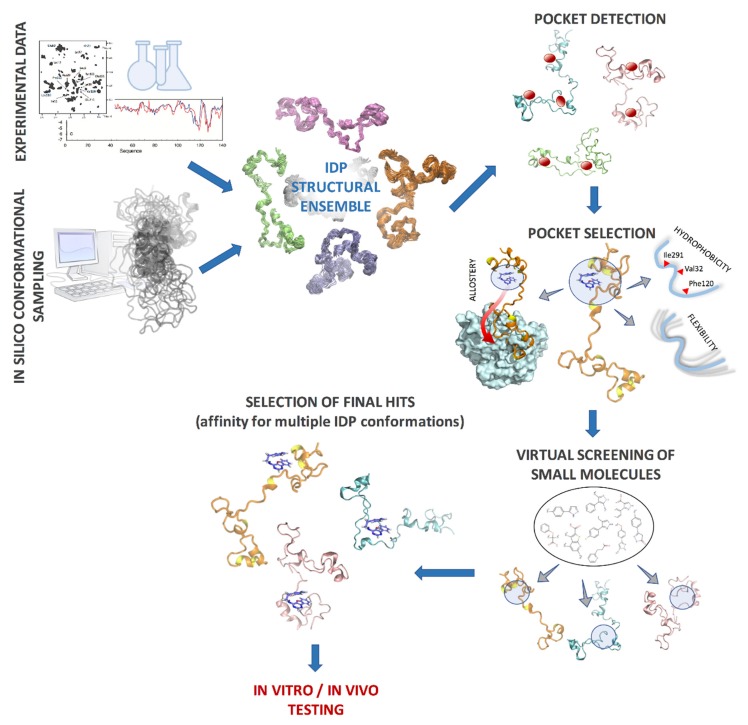
Example computational protocol for identifying drug candidates for IDPs; the IDP structural ensemble is clustered into representative conformations and druggable pockets are identified in each conformation; these pockets are further filtered by criteria such as hydrophobicity, backbone flexibility, and allosteric communication with functional sites. Virtual screening is performed in each pocket and final hits are selected based on high affinity in multiple IDP conformations.

Intrinsically disordered proteins are also promising targets for developing allosteric modulators, since conformational flexibility has been shown to enhance allostery in IDPs and bacterial flagellar switch [142,143,144,145]. Methods like ALLOSTEER, which can identify pockets that are both druggable and allosterically active, can immensely benefit IDP drug discovery [146]. Using ALLOSTEER, it is possible to identify allosterically active pockets that are distant from the protein–protein interface and the binding site residues that are critical for allostery [147]. Drugs binding to such pockets can modulate the binding of the IDP to the partner protein, while not having to compete directly with the partner protein by binding at the interface. A computational protocol for designing small molecules for an IDP therefore could start with a protein structural ensemble obtained from experimental data and computational sampling, followed by clustering of conformations and identification of druggable pockets in each cluster representative, using methods such as FindBindSite [148] and FPOCKET [149], and narrowed down using design principles such as structural rigidity, hydrophobicity, and allostery. Then, conventional HTVS methods can be used to screen small molecule libraries in each pocket, and compounds which show low binding energies in multiple conformations can be selected as potential drug candidates (Figure 4). Such protocols are likely to improve with future insights gained in the interaction between IDPs and small molecules.

## 6. Concluding Remarks

Compared to folded proteins, the energy landscape of an IDP is seemingly more complex with multiple populated basins at physiological conditions. With folded proteins, the challenges in computationally exploring the energy landscape come from the free-energy barriers separating the intermediate states along the folding pathway and the difficulty in energetically distinguishing the native ensemble from decoys. In addition to this, the challenges with IDPs are the vast degrees of freedom and the intricate balance between the inter-protein and solvent interactions that govern structural equilibrium. Methods that use experimental data to select a subset of conformations from a statistical coil ensemble do not cover all possible conformations of an IDP, which can be stellar even for a short polypeptide chain. A major concern is that transient or sparsely populated structures that do not significantly contribute to the statistical average properties may be missed. These transient conformations could be nevertheless critical for partner protein recognition or inhibitor binding. The other question is regarding the uniqueness of the structural ensemble that is obtained by fitting to the experimental NMR data, the so called degeneracy issue [22,150]. Normally, a large number of experimental restraints are needed to uniquely reproduce an IDP ensemble using computational sampling. Such experimental data are rarely available, leading to potentially ambiguous results from the fitting [151]. Thus, careful cross-validation of the predicted ensembles is needed, possibly using alternative experimental methods such as site-directed mutagenesis. The relevance and accuracy of the experimental data itself is also a matter of concern, since experimental measurements often require modification of the protein by attaching probes and labels. Given the fact that, for many IDPs, the conformational equilibrium is highly sensitive to post-translational modifications such as phosphorylation, it is debatable to what extent NMR probes affect the IDP ensembles [46]. De novo methods like MD and enhanced sampling algorithms, that model protein dynamics using physics-based approaches, can solve some of the aforementioned issues, but these methods have their own challenges due to limited computational power and inaccurate force fields. Analyzing recent literature, it is clear that more improvement is needed to accurately describe the energetics of IDPs, including the balance between secondary structure formation and solvent interaction. The heterogeneous solvent environment of IDPs makes it harder to model the chemical nature of the polypeptide chain during dynamics simulations, such as polarization effects and protonation states of charged residues and histidines, which can change more rapidly compared to the folded protein environment. Moreover, the expanded structures of IDPs require larger simulation boxes than folded proteins, which is a hindrance towards fully sampling the IDP dynamics using atomistic simulations. Thus, a multiscale approach is better suited for conformational sampling of IDPs, where an initial ensemble of backbone conformations obtained through coarse grain methods can be further refined using finer resolution algorithms, giving detailed information such as the orientation of residue sidechains or transition barriers. These ensembles must be validated against available experimental data and compared with the ensembles obtained through random coil algorithms. The available public databases of IDP ensembles can largely aid in the development and improvement of computational techniques for exploring IDPs [152,153].

In recent years, multiple experimental and computational studies have given insight into the binding mechanisms of IDPs, such as conformational transitions upon binding and the associated kinetics. However, more research is needed in this field, especially since protein–protein binding is a complex topic that is not fully understood, even for folded proteins. It is not clear, for example, how IDPs recognize their partner proteins with such high specificity, despite a lack of specific inter-residue interactions at the binding interface, and how dynamics plays its role in the binding specificity. Molecular modeling and simulation studies can be complemented by co-evolutionary analysis of IDPs and their partners. Co-evolutionary analysis has been heavily explored for folded proteins [154,155], and is just now being studied for IDPs [156]. Such insights will be invaluable in the rational design of therapy targeting IDPs, i.e., small molecules and peptides to disrupt binding and aggregation.
